# Cochlear Implants after Meningitis and Otosclerosis: A Comparison between Cochlear Ossification and Speech Perception Tests

**DOI:** 10.3390/jpm14040428

**Published:** 2024-04-18

**Authors:** Pauliana Lamounier, Natalia Carasek, Valeria Barcelos Daher, Claudiney Cândido Costa, Hugo Valter Lisboa Ramos, Sergio de Castro Martins, Alda Linhares de Freitas Borges, Lucas Alves Teixeira Oliveira, Fayez Bahmad Jr

**Affiliations:** 1 Department of Otolaryngology, Center of Rehabilitation and Readaptation Dr Henrique Santillo (CRER), Goiania 74653-230, Brazil; paulianalamounierorl@gmail.com (P.L.); valeriabdaher@gmail.com (V.B.D.); claudineyccosta@gmail.com (C.C.C.); hvlramos@gmail.com (H.V.L.R.); sergio.martins@ueg.br (S.d.C.M.); alda.lfb@gmail.com (A.L.d.F.B.); 2 Department of Health Sciences, University of Brasilia, Brasilia 70910-900, Brazil; ncarasek@gmail.com (N.C.); lucasat.oliveira@gmail.com (L.A.T.O.); 3 Otorhinolaryngology Department, Universidade Estadual de Goiás (UEG), Itumbiara 75536-100, Brazil

**Keywords:** cochlear implants, otosclerosis, meningitis, speech perception

## Abstract

(1) Background: Performance after Cochlear Implantation (CI) can vary depending on numerous factors. This study aims to investigate how meningitis or otosclerosis can influence CI performance. (2) Methods: Retrospective analysis of CI performance in patients with etiological diagnosis of meningitis or otosclerosis, comparing the etiologies and analyzing the image findings, along with electrode array insertion status and technique. (3) Results: Speech recognition in CI patients with otosclerosis improves faster than in patients with meningitis. Other features such as radiological findings, degree of cochlear ossification, surgical technique used and total or partial insertion of electrodes do not seem to be directly related to speech recognition test performance. (4) Conclusions: Patients should be warned that their postoperative results have a strong correlation with the disease that caused their hearing loss and that, in cases of meningitis, a longer duration of speech–language training may be necessary to reach satisfactory results.

## 1. Introduction

Meningitis is defined as the inflammation of the meninges that is most often caused by various bacterial and viral pathogens. It is associated with high rates of mortality and morbidity [1]. The most common sequela after bacterial meningitis is hearing loss. This is particularly common after pneumococcal meningitis, where as many as 30% of patients suffer from hearing loss [2]. It usually presents as bilateral, deep, symmetrical, sensorineural hearing loss and is more frequent in males and in children under 5 years old. The most common presentation is hearing loss that occurs early, within 2 days of the onset of rapidly progressive bacterial meningitis; however, it can occur up to 10 to 12 years after the onset of the disease [3].

The route of infection is usually through bacterial or toxin invasion from the meningeal space into the inner ear via the internal auditory canal and cochlear aqueduct (a small canal that contains the perilymphatic duct, communicating the subarachnoid space and the tympanic ramp of the cochlea’s basal turn). Bacteria and leukocytes reach the perilymphatic space, leading to a proliferation of fibroblasts, fibrosis and ultimately the ossification of the cochlea [3,4]. The final stage of this process results in a cochlea with stenosis and calcification. The progression of infection can also reach fibers of the spiral ganglion, through the modiolus and cochlear nerve or brainstem, leaving fewer functional neural fibers remaining. The clinical consequence may be a patient with deep sensorineural hearing loss [4,5,6].

Meningitis is also capable of generating cortical necrosis, as well as hippocampal apoptosis [7]. Up to 24% of cases can generate focal neurological deficits, such as hemiparesis, cranial neuropathies and memory impairment [8]. These central changes and the reduction in spiral ganglion neurons in the cochlea are bad predictors for the effectiveness of the cochlear implant (CI). Better hearing results are associated with less fibrous occlusion of the perilymphatic space [7].

Otosclerosis is an early adult-onset disease and a common cause of hearing loss due to the bone remodeling of the otic capsule [9]. It is characterized by progressive dysplasia of the otic capsule with bony resorption, vascular proliferation, and sclerotic new bone formation [10]. The extent and location of this aberrant remodeling are determinant to the type of deafness the patient may present, ranging from conductive hearing loss due to stapes footplate fixation (the most common presentation), to a sensorineural hearing loss secondary to the invasion of the cochlear endosteum, or a combined form of both [9,10]. The involvement of retrofenestral structures, such as the cochlear endosteum, with compact bone and vascular neoformation results in labyrinthitis ossificans. Long periods of auditory deprivation in this type of insidious hearing loss can also lead to the degeneration of spiral ganglion cells [9]. Cochlear implantation is the main form of auditory rehabilitation for those patients with profound sensorineural hearing loss who do not benefit from hearing aids, even in cases of far-advanced otosclerosis, demonstrating significant gains in speech-discrimination scores [10].

As the CI electrode insertion is performed on the tympanic scale of the cochlea, ossification or some degree of fibrosis in its lumen—as may happen in meningitis or otosclerosis—can represent a challenge for the surgery or its audiological outcomes [9,11,12]. Previous studies [13] suggest that CI in cochlear ossification is feasible despite surgical challenges and modifications. However, when it comes to auditory outcomes, the literature disagrees [13,14]. Therefore, this study aims to investigate how meningitis or otosclerosis can influence CI performance and its relation to a difficulty of electrode placement caused by cochlear ossification or the involvement of neural fibers.

## 2. Materials and Methods

Retrospective analysis of data from cochlear implant cases in patients diagnosed with meningitis or otosclerosis who met the eligibility criteria.

### 2.1. Inclusion Criteria

- Adult individuals with post-lingual severe to profound hearing loss whose etiologic diagnosis was meningitis or otosclerosis;
- Unilateral cochlear implant users;
- Effective use of the device for at least 1 year;
- In regular speech therapy;
- Available preoperative imaging tests.

### 2.2. Exclusion Criteria

- Subjects under 18 years of age;
- History of neurological or cognitive alterations (that could interfere in the evaluation);
- Abnormal electrode impedances in all modes of stimulation;
- First language is not Brazilian Portuguese.

Eight subjects with deep post-lingual hearing loss due to meningitis and eight due to otosclerosis, totaling sixteen individuals, were included in the study. Comparison of performance with cochlear implants in those two groups was made. The subjects were evaluated by speech perception tests through auditory discrimination tests with a list of sentences and a list of disyllables. The tests were applied at 3 different time points: preoperative, after 6 months and after 1 year of activation of the cochlear implant.

All selected patients underwent the speech perception test in a cabin of dimensions 2 × 2 m, using the same audiometer (Interacoustics, AC 33, calibration A0653006/19) connected to a speaker via an Orlandi acoustic amplifier. Sound stimuli of the warble type were presented in a sound field at frequencies from 500 to 8000 Hz. The speech perception test was carried out through a list containing 10 sentences with 50 phonetically balanced characters developed and validated by the Center of Audiological Studies (CPA) in Bauru-SP, Brazil, with the correctness of each character in the value of 2 scores (2%), and through a list containing 25 phonetically balanced disyllabic words developed and validated by the CPA, with the correctness of each word in the value of 4 scores (4%).

The speech perception test was performed using the list of sentences recorded on a CD by an announcer with a male voice. The sentences were distributed, maintaining constant 10 s intervals between the end of one sentence and the beginning of the next and allowing time for the patient to respond and prepare to listen to the next sentence. The audiometer attenuator was fixed, and the speech was presented through a loudspeaker positioned at 45° azimuth of the patient, according to the calibration of the free field, and was controlled by 2 examiners. The room had a reduced noise level. While completing the tests, the patient was subjected to open-set stimulation (without visual clues). Our team double-checked at each step that the patient understood the content of the tests. The average of 500 Hz to 4 Hz was used for calculations.

A comparison between the etiology of meningitis vs. otosclerosis was conducted, analyzing the findings of Computed Tomography (CT) of the mastoid and Magnetic Resonance Imaging (MRI) of the ear (such as signs of ossification, cochlear nerve hypoplasia and signs of otosclerosis) along with electrode array insertion status (total or partial, via cochleostomy or round window) in relation to speech perception. The objective was to verify whether there was a significant difference of the perception test results in those described variables. The analysis of the imaging was performed by a radiologist who was not aware of the patients’ diagnosis.

The existence of a correlation between the perception test and the variables of length of hearing-aid use and duration of deafness were also analyzed.

### 2.3. Ethical Considerations

This study was conducted in accordance with the Declaration of Helsinki and informed consent was obtained from the patients to write and submit this paper, alongside with the disclosure of images and test results. The project was approved by the Research Ethics Committee 5082—Leide das Neves Ferreira (LNF), which belongs to the research institution Centro de Reabilitação e Readaptação dr Henrique Santilo (CRER), registered through the Plataforma Brasil (a Brazilian national and unified database of research records involving human beings—CEP/Conep system), under number 36929420.1.0000.5082. Ethical approval date from CEP/CONEP was 13 September 2021. Patients’ information was anonymized and the data securely stored.

### 2.4. Statistical Analysis

Data were exported and handled in Excel for further processing using the Statistical Package for Social Science (SPSS) [15] program (version 21.0) for Windows. Categorical variables are presented as absolute values (f) and percentages (%). Continuous variables are presented as the mean ± standard deviation and median (95% CI). The Wilcoxon test [16] was used to compare the speech perception test with disyllables and in the sentences regarding the preoperative, 6 months postoperative and 1 year postoperative time frames in both etiologies (meningitis and otosclerosis).

Data from CT scans and MRI from the patients were compared and correlated to the information about electrode insertion (total or partial and via cochleostomy or round window). Mann–Whitney test [17] was used to verify whether there was a significant difference in the perception test according to these variables.

Linear regression was used to verify the existence or non-existence of a correlation between the perception test and the following variables: length of use of individual sound amplifiers and deafness time. Fisher’s exact test [18] was used to verify whether there was a significant difference between the results of CT and MRI findings in relation to the insertion of the electrode (total or partial, via cochleostomy or round window). For all tests, a 95% confidence level (*p* < 0.05), was considered significant.

## 3. Results

Our study included 16 patients, 8 diagnosed with otosclerosis and 8 with meningitis. Half of the patients were female and the other half male. Regarding the brand of cochlear implants, the otosclerosis group had two patients with Advanced Bionics (Naida processor, Hi Res 90 K, 1 J electrode), three patients with MED-EL (two Sonata processors, standard electrode and one Concerto processor, standard electrode), and three patients with Neurelec (Digisonic SP) devices. In the meningitis group, four patients were implanted with MED-EL (three Sonata processors, standard electrode and one Concerto processor, compressed electrode), one patient with Cochlear (Freedom, CI 422 electrode) and three patients with Neurelec (Digisonic SP). The choice of brand in our service happens through a rotation, so that, at the end of the year, the same number of implants of each brand is performed.

Nine of the patients presented some temporal bone tomography or resonance abnormalities. Thirteen patients had total electrode insertion, but in the other three, only a partial insertion of electrodes was feasible. The mean age at time of surgery was 53.9 years old (±10.2) for meningitis and 56.9 years (±6.8) for otosclerosis. The average duration of deafness was 28.4 years (±7.5) for otosclerosis and 16.9 years (±20.9) for meningitis. The mean length of hearing aids usage was 12.9 years (±14.9) for meningitis and 14.2 (±12.1) for otosclerosis. The age of onset of hearing loss was 37.0 (±24.9) years for patients with meningitis and 28.6 (±10.9) years for patients with otosclerosis. These findings are synthesized in Table 1.

**Figure 1:**
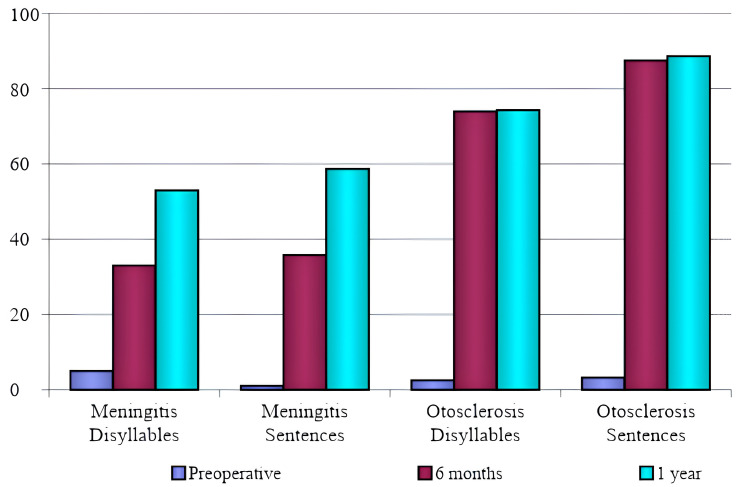
Average percentage of correct answers on the speech perception tests by etiology.

The parameters of the speech perception test results are listed in Figure 1, and the comparison between meningitis and otosclerosis is shown in Table 2 and Table 3. The comparison showed that, 6 months postoperatively, speech recognition in patients with otosclerosis was better than in meningitis patients, which was statistically significant.

Regarding the first-year evaluation of otosclerosis patients, there was a statistically significant difference in the sentence test, but not in the dissyllable test. There was improvement in the speech recognition test for meningitis patients at 6 months and 1 year after activation. Concerning the previous length of hearing aid use, there was no significant correlation between the results of speech recognition, disyllables or sentences at 6 months and 1 year in both meningitis and otosclerosis groups (*p* < 0.05), as shown in Table 4.

Deafness duration had a significant negative correlation with the results of speech recognition, which means that a shorter duration of deafness resulted in better speech recognition outcomes. Those results happened in the meningitis group with the disyllable test at 6 months post activation and in the otosclerosis group after 1 year (Table 5).

**Figure 2:**
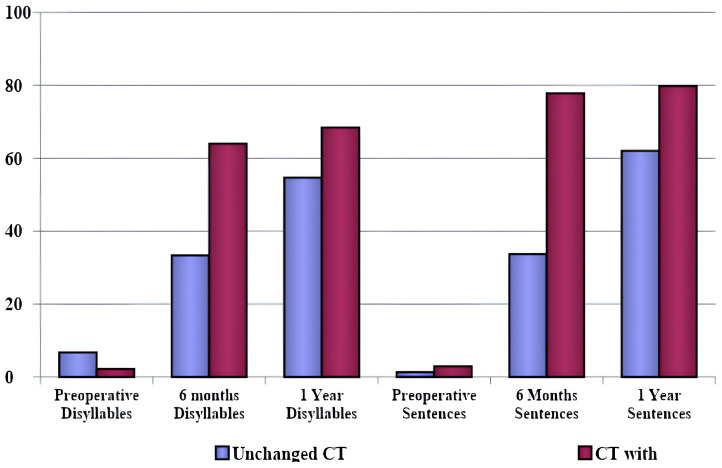
Comparison between patients who presented and those who did not present changes in computed tomography for speech perception tests.

**Figure 3:**
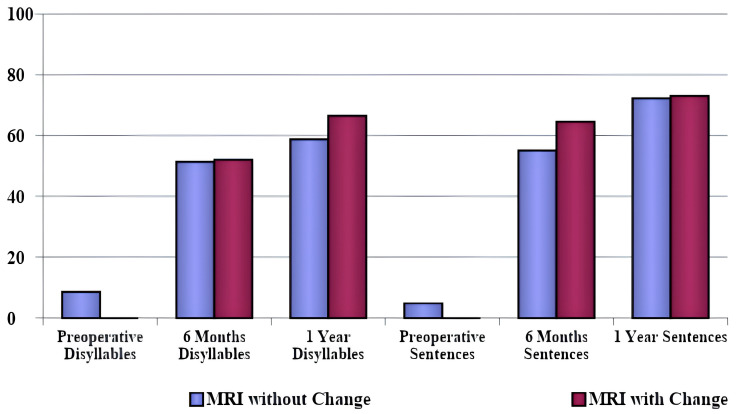
Comparison between patients who presented and those who did not present changes in magnetic resonance imaging for speech perception tests.

The comparison between patients who presented radiological changes and those who did not is shown in Figure 2 for CT scans and in Figure 3 for MRIs. Table 6 shows the radiological changes presented by the patients. The comparison between speech perception tests for patients who presented changes in CT scans and those who did not showed significantly higher scores at 6 months in the group with radiologic alterations (*p* = 0.026). The same correlation did not occur 1 year postoperatively.

In the meningitis group, 25% of patients had partial insertion and 75% had complete insertion. In the otosclerosis group, 12.5% had partial insertion, and 87.5% had complete insertion; however, this difference was not statistically significant (*p* = 0.500). The comparison between partially inserted electrodes and completely inserted ones is shown in Table 7. The analysis of active electrodes intraoperatively, at 6 months and at 1 year, also did not show a significant difference.

Two patients in the meningitis group had partial insertion, of whom one had abnormal MRI results and neither had abnormal CT scans. In the otosclerosis group, only one patient had partial insertion, along with changes only in the MRI results. Due to the low number of patients with partial insertion, comparison of speech recognition was not possible for those subjects. The aided Pure Tone Average (PTA) of all patients, confirming the functionality of the system, is listed in Table 8. There was no significant difference between groups.

## 4. Discussion

The performance after cochlear implantation can vary depending on numerous factors. The etiology of the hearing loss, its duration, use of hearing aids, age of onset, CT and MRI findings and electrode insertion are possible variables impacting on the hearing outcomes [19]. One of our goals was to determine whether performance on these adult patients could be linked to the etiology of the disease. The etiologies of meningitis and otosclerosis were thoroughly characterized in our patients, ensuring a well-defined diagnosis. Furthermore, an assessment on whether the post-implant results in these diseases are more related to the etiology of the disease or to the presence of radiological changes was made. Despite our small sample, it showed statistically significant results.

In meningitis, the dissemination of the infection may occur through the cochlear aqueduct—which has a direct connection with the subarachnoid space—or through the endolymphatic sac, causing complete or partial destruction of the sensory receptors [20]. In otosclerosis, bone demineralization in the otic capsule could also be related to variable performances in speech recognition after a cochlear implant [21].

The analysis of speech recognition tests (disyllables and sentences) in the meningitis group showed a statistically significant improvement when comparing the preoperative test with the 6-month evaluation, the preoperative period with 1-year postoperative results, and the 6-month postoperative period with 1-year test results. In the otosclerosis group, there was a statistically significant improvement in speech recognition comparing the preoperative period with both the 6-months and the 1-year postoperative period. In the comparison between the postoperative period of 6 months and 1 year, there was an improvement, but not statistically significant. We conclude from these data that the improvement in speech recognition tests observed in the postoperative period of otosclerosis is more significant within the first 6 months, when the patients reach a stable plateau of speech comprehension which does not change in a statistically significant way for up to 1 year.

The comparison between the two groups showed that, after 6 months, speech recognition in patients with otosclerosis was better than in meningitis patients, which was statistically significant. Regarding the 1-year test, there was a statistically significant difference in the sentence test in otosclerosis patients but not in the dissyllable test. There was improvement in the speech recognition test in meningitis patients at 6 months and 1 year.

Many factors may potentially influence one’s comprehension of speech with a cochlear implant. Aside from the duration of deafness, there was no agreement among studies on which factors have the greatest bearing on speech recognition. Labyrinthitis ossificans may result in segmental loss of spiral ganglion cells in regions of cochlear ossification, and surviving auditory neurons suitable for stimulation may simply be too small to provide useful speech recognition [22]. Furthermore, it is imperative to remember about the possible presence of neurological sequelae (at the central nervous system level), since meningitis may affect the central auditory pathway independently from cochlear pathology [23,24].

Regarding the insertion of the electrode, in the meningitis group, 25% had partial insertion and 75% had total insertion; in the otosclerosis group, 12.5% had partial insertion and 87.5% total insertion. However, this difference was not statistically significant (*p* = 0.500). There was also no statistically significant difference in the PTA between the two groups. No difference was observed also regarding insertion by cochleostomy or round window when related to the speech perception tests. The analysis of the active electrodes in the intraoperative period, at 6 months and 1 year, also did not present a significant difference.

The comparison between speech perception tests in patients who presented and those who did not present changes in the MRI tests (regardless of etiology) did not show a statistically significant long-term difference. Patients who presented alterations in the CT scans performed worse in the speech perception tests than those who had normal CTs, showing a significantly lower score on the sentence speech perception test at 6 months (*p* = 0.026), but not after 1 year postoperative. Therefore, we infer that radiological findings in both CT and MRI scans are not directly linked to long-term poorer performance on speech perception tests. This association was observed regarding the etiological diagnosis (meningitis or otosclerosis), but not the degree of cochlear ossification in the images. Changes in the images were more prevalent in the otosclerosis group, as shown in Table 6, and this was the group that presented better speech recognition thresholds.

The degree of neural survival is an important factor in the ability to process speech stimuli. Therefore, a more accurate determination of the neural structures that can be stimulated with electrical impulses would be quite useful because it is assumed that the survival of ganglion cells and other elements of central auditory pathways might constitute one of the causes of the variable speech recognition performance found among individuals with cochlear implants [19,25]. Vashishth [13] and Wang [26] demonstrated that diseases such as meningitis may affect the central auditory pathways, resulting in poorer outcomes regardless of ossification. Our mean scores in the vowel, word, sentence and comprehension categories improved significantly with time and these results agree with the ones found in previous studies [13,26]. In our study, patients diagnosed with meningitis took more time to reach levels of speech recognition that were similar to those of patients with otosclerosis; in this group, speech recognition had already been reached by 6 months.

Marshall et al. [27] showed no significant difference in post-implantation sentence test scores at 6 months and at 1 year between otosclerotic and non-otosclerotic patients. A definite association between the extent of ossification and auditory outcomes has not been demonstrated according to Senn et al. [28]. Nichani et al. [29] had encouraging results in their study and showed that patients with ossified cochlea might express satisfactory categories of auditory performance (CAP) scores, similar to the CAP scores of patients with non-ossified cochlea.

Kraaijenga et al. [30] found a significant negative correlation between meningitis and otosclerosis and speech performance scores post-implant. They showed that deafness due to bacterial labyrinthitis results in a significantly worse performance than patients with other etiologies of hearing loss. These data are similar to our findings. By removing all patients with partial insertions in the sensitivity analysis, they showed that poorer speech perception in patients with meningitis and otosclerosis is not only due to partial insertions but also due to the disease itself [30].

The duration of deafness among patients with meningitis was shorter than among patients with otosclerosis in our study. Matterson et al. [31] found that, in patients with otosclerosis, the duration of deafness in the implanted ear and the age at implantation are both negatively correlated with post-implantation speech perception outcomes at 3 months but cease to be significant by 6 months postoperatively. Other studies [32] have demonstrated that the duration of bilateral deafness is a stronger predictor of speech perception. Our data showed no statistically significant difference in the duration of hearing-aid use regarding the speech perception test, but deafness duration showed a significant negative correlation to the speech perception thresholds. However, when comparing the meningitis and otosclerosis groups with each other, it was observed that patients with otosclerosis had better results in speech perception tests, despite having a longer period of deafness.

Blamey et al. [33] showed that, even though traditionally considered a factor adversely associated with post-implantation speech perception outcomes, the effect of a long duration of deafness has become a less important factor over the past years. The negative effect of a long duration of severe to profound hearing loss was less important in the new data than in past studies, but patients with longer durations of deafness were still less likely to improve with CI experience; which means that a duration of severe deafness of more than 40 years and an age at implantation of more than 75 years still result in negative correlations with outcomes. Other authors [30] found that the highest variability was accounted for by age at onset of deafness as a predictor instead of the more reported duration of deafness.

Matterson et al. [31] concluded that patients with implants in the ear with a longer duration of deafness should be advised that it may take up to 6 months to achieve speech perception results similar to what might be expected for patients with shorter hearing deprivation. In our study, we observed that the improvement in speech comprehension in patients with otosclerosis was faster than in patients with meningitis, even if they had a greater duration of deafness. This might not be the case if we were comparing patients with otosclerosis with another group of patients who did not present the neural damage caused by meningitis [31]. The neural damage caused by meningitis outweighs the greater duration of deafness characteristic of otosclerosis in terms of the results of speech perception tests in our sample.

Vashishth et al. [13] concluded in their review about cochlear ossification that although otosclerotic patients may initially perform better on word scores, no significant differences remained 1 year post implantation compared with non-otosclerotic pathologies. The improvement of comprehension in patients with otosclerosis is faster, since neural damage is probably more significant in meningitis, delaying improvement in recognition tests, which appears later after speech–language training.

The cochlear implant can bring an important improvement in patients’ quality of life. As our population is aging, there is an increasing need for strategies of hearing rehabilitation, such as hearing aids and cochlear implantation [34]. The increasing life expectancy results in adults and elderly individuals with hearing loss being a meaningful part of the economically active population for a considerable period of time and, therefore, they must be properly rehabilitated to enhance performance. CI, along with speech therapy, can provide patients with the opportunity to greatly improve their speech production and communication skills.

### Study Limitations

- The number of subjects in this study is small, therefore the results can suggest but not confirm the findings.
- The cognitive function of the patients was not analyzed in a standardized manner, but a history of neurological or cognitive alterations was considered an exclusion factor.

## 5. Conclusions

Speech comprehension after CI can vary greatly. Our results suggest that speech recognition in CI patients with otosclerosis improves faster than in patients with meningitis. Other features such as radiological findings, degree of cochlear ossification, surgical technique used and total or partial insertion of electrodes do not seem to be directly related to speech recognition tests performances. Patients should be warned that their postoperative results have a strong correlation with the disease that caused their hearing loss and that in cases of meningitis, a longer duration of speech–language training may be necessary to reach satisfactory thresholds.

## Figures and Tables

**Table 1:** Parameter of variables regarding groups.

Variable	Meningitis		Otosclerosis		*p*
	Mean	SD	Mean	SD	
Age	53.9	10.2	56.9	6.8	0.645
Duration of deafness	16.9	20.9	28.4	7.5	0.207
Age of onset of hearing loss	37.0	24.9	28.6	10.9	0.600
Length of hearing aid use	12.9	14.9	14.2	12.1	0.574

Test used: Mann–Whitney.

**Table 2:** Comparison between speech perception tests.

Comparison (Test)	Disyllables	Sentences
Meningitis		
Preoperative × 6 months	0.017 *	0.018 *
Preoperative × 1 year	0.012 *	0.012 *
6 months × 1 year	0.025 *	0.018 *
Otosclerosis		
Preoperative × 6 months	0.012 *	0.012 *
Preoperative × 1 year	0.018 *	0.017 *
6 months × 1 year	0.916	0.916

Test used: Wilcoxon. * *p* value < 0.05

**Table 3:** Comparison between patients with meningitis and those with otosclerosis in speech perception tests.

Comparison (Test)	Meningitis	Otosclerosis	*p*-Value
Test in preoperative			
Disyllables	5.0 ± 14.1	2.5 ± 7.1	0.959
Sentences	1.0 ± 2.8	3.2 ± 9.2	0.959
Test in postoperative (6 months)			
Disyllables	33.0 ± 12.8	74.0 ± 20.3	0.003 *
Sentences	35.8 ± 20.3	87.5 ± 13.9	0.001 *
Test in postoperative (1 year)			
Disyllables	53.0 ± 24.1	74.3 ± 14.4	0.081
Sentences	58.7 ± 25.2	88.6 ± 11.6	0.005 *

Test used: Mann–Whitney. * *p* value < 0.05

**Table 4:** Correlation between the use of hearing aids and speech recognition in groups.

Variable	R^2^	B	*p*
Meningitis			
Disyllable 6 months	0.277	−0.404	0.180
Sentences 6 months	0.132	−0.443	0.376
Disyllables 1 year	0.014	−0.169	0.783
Sentences 1 year	0.020	−0.216	0.736
Otosclerosis			
Disyllables 6 months	0.171	−0.694	0.309
Sentences 6 months	0.062	−0.289	0.550
Disyllables 1 year	0.231	−0.625	0.275
Sentences 1 year	0.231	−0.505	0.275

Test used: Linear regression.

**Table 5:** Correlation between deafness time and speech recognition in groups.

Variable	R^2^	B	*p*
Meningitis			
Disyllable 6 months	0.524	−0.443	0.042 *
Sentences 6 months	0.166	−0.395	0.317
Disyllable 1 year	0.034	−0.214	0.660
Sentences 1 year	0.001	0.044	0.931
Otosclerosis			
Disyllable 6 months	0.227	−1.282	0.233
Sentences 6 months	0.390	−1.157	0.098
Disyllable 1 year	0.626	−1.789	0.034 *
Sentences 1 year	0.451	−1.228	0.099

Test used: Linear regression. * *p* value < 0.05.

**Table 6:** Description of radiological changes.

Patient	Etiology	Computed Tomography	Magnetic Resonance Imaging
1	Otosclerosis	Bilateral basal spiral ossification	Bilateral basal spiral ossification
2	Otosclerosis	Bilateral otosclerosis optic capsule	Bilateral otosclerosis optic capsule
3	Meningitis	Normal	Normal
4	Meningitis	Normal	Normal
5	Meningitis	Normal	Normal
6	Meningitis	Bilateral malformation of the stapes (1 crura)	Bilateral cochlear hypoplasia
7	Otosclerosis	Bilateral otosclerosis with normal lumen	Normal
8	Otosclerosis	Bilateral basal spiral ossification	Bilateral reduction in basal spiral lumen
9	Meningitis	Bone demineralization of the optic capsule and fissula ante fenestram	Normal
10	Otosclerosis	Normal	Bilateral cochlear hypoplasia
11	Meningitis	Normal	Left intracanalicular Schwannoma
12	Meningitis	Bilateral cochlear and fenestral otosclerosis	Bilateral pericochlear impregnation
13	Otosclerosis	Normal	Bilateral partial ossifying labyrinthitis
14	Meningitis	Pericochlear bilateral bone demineralization	Pericochlear signal alteration with bilateral basal turn lumen reduction
15	Otosclerosis	Normal	Bilateral partial ossifying labyrinthitis
16	Otosclerosis	Fenestral and retrofenestral bilateral otosclerosis	Fenestral and retrofenestral bilateral otosclerosis

**Table 7:** Data of the comparison between partially inserted electrodes with the normal insertion.

Electrodes Insertion	Meningitis	Otosclerosis	*p*-Value
Partial	2/25.0%	1/12.5%	0.500
Normal	6/75.0%	7/87.5%	

Test used: Fisher.

**Table 8:** PTA of the patients confirming the fitting of the system.

Pure Tone Average (PTA)	Meningitis	Otosclerosis	*p*-Value
250 Hz ^1^	36.2 ± 7.90	35.00 ± 10.69	0.914
500 Hz	35.62 ± 6.23	34.37 ± 10.15	0.771
1000 Hz	30.62 ± 5.62	31.87 ± 7.52	0.713
2000 Hz	32.50 ± 7.55	31.25 ± 11.87	0.805
3000 Hz	33.75 ± 6.94	34.37 ± 11.47	0.897
4000 HZ	36.25 ± 5.82	36.25 ± 9.16	1.000
6000 HZ	35.62 ± 5.62	37.50 ± 10.69	0.670
8000 Hz	41.87 ± 16.88	43.75 ± 10.60	0.794

Test used: t-Student, ^1^ Mann–Whitney.

## Data Availability

The original contributions presented in the study are included in the article; further inquiries can be directed to the corresponding author. The raw data supporting the conclusions of this article will be made available by the authors on request.
